# Fish Feed Intake, Feeding Behavior, and the Physiological Response of Apelin to Fasting and Refeeding

**DOI:** 10.3389/fendo.2021.798903

**Published:** 2021-12-15

**Authors:** Daniel Assan, Yanlin Huang, Umar Farouk Mustapha, Mercy Nabila Addah, Guangli Li, Huapu Chen

**Affiliations:** ^1^ Fisheries College, Guangdong Ocean University, Zhanjiang, China; ^2^ Guangdong Research Center on Reproductive Control and Breeding Technology of Indigenous Valuable Fish Species, Guangdong Ocean University, Zhanjiang, China; ^3^ Guangdong Provincial Engineering Laboratory for Mariculture Organism Breeding, Zhanjiang, China; ^4^ Guangdong Provincial Key Laboratory of Pathogenic Biology and Epidemiology for Aquatic Economic Animals, Zhanjiang, China; ^5^ Southern Marine Science and Engineering Guangdong Laboratory, Zhanjiang, China; ^6^ Department of Fisheries and Aquatic Resources Management, Faculty of Bioscience, University for Development Studies, Tamale, Ghana

**Keywords:** apelin, feeding behavior, feed intake, fish, orexigenic

## Abstract

Feed is one of the most important external signals in fish that stimulates its feeding behavior and growth. The intake of feed is the main factor determining efficiency and cost, maximizing production efficiency in a fish farming firm. The physiological mechanism regulating food intake lies between an intricate connection linking central and peripheral signals that are unified in the hypothalamus consequently responding to the release of appetite-regulating genes that eventually induce or hinder appetite, such as apelin; a recently discovered peptide produced by several tissues with diverse physiological actions mediated by its receptor, such as feed regulation. Extrinsic factors have a great influence on food intake and feeding behavior in fish. Under these factors, feeding in fish is decontrolled and the appetite indicators in the brain do not function appropriately thus, in controlling conditions which result in the fluctuations in the expression of these appetite-relating genes, which in turn decrease food consumption. Here, we examine the research advancements in fish feeding behavior regarding dietary selection and preference and identify some key external influences on feed intake and feeding behavior. Also, we present summaries of the results of research findings on apelin as an appetite-regulating hormone in fish. We also identified gaps in knowledge and directions for future research to fully ascertain the functional importance of apelin in fish.

## 1 Introduction

Food is one of the foremost expenses of intensive fish farming, which fish farmers need to pay much attention to. Its availability in quantity and quality is significant for the appropriate growth and reproduction of fish (1). Feeding as determined by Metcalfe and colleagues (2) plays a vital role in animal life-sustaining activities. Evidence has it that, the regulation of feed intake, as in mammals, is well conserved in vertebrates, including some fish species (3, 4). The optimization of food intake can lead to enhanced growth and body composition, with increased food conversion efficiency and reduced nutrient losses, which are major objectives in intensive fish farming (1).

The intake of feed is known to be regulated by complex interactions between the brain and peripheral appetite-regulating hormonal factors, including apelin (5). As indicated by Volkoff and colleagues (1), when the endocrine mechanisms controlling food intake in fish is understood, it will not only lead to the explicit modifications in fish-holding situations and feeding approaches such as temperature and time of feeding respectively but rather, it will also help to develop new procedures to improve food conversion efficiency as well as aquaculture growth.

In the past years, less attention has been given to apelin in the regulation of feed intake in vertebrates. With its uncertain role in mammals as a feed intake regulator, apelin has been identified to play an orexigenic role in vertebrates such as fish. It aids in several regulation of biological activities in fish, which most importantly includes the regulation of food consumption. The goal of this review is twofold; firstly, to examine the recent advances in our understanding of the feeding behavior, focusing more on dietary selection and preference of fish as well as analyzing the influence that some external factors have on feed intake and behavior. Secondly, we gather information from previous research studies on apelin, categorizing its specific role in fish as an appetite-regulating hormone and identifying gaps in knowledge and directions for future research regarding this important topic.

## 2 Feed Intake and Feeding Behavior in Fish

The result of food intake is the alteration that lies between starvation, craving, and satiation. Starvation is the physiological necessity for food, including a strong incitement to feeding behavior; looking for food and consuming it. Satiation is the physiological and rational sense of “fullness” that happens after food intake whiles appetite or craving, on the other hand, is the desire to eat, which is commonly related to the material (find, fragrance, taste) perceptiveness of the food to be consumed (6).

Feed is among the most authoritative signals outside the fish’s body that can arouse feeding behavior and growth (7–9). Its readiness and composition exert a key control of these processes, by acting principally on the hormones responsible for their endocrine control (9). Some central and peripheral appetite regulators in fishes are affected by a single meal, showing per-prandial fluctuations in their expression and/or secretion levels. Such changes in fishes have been identified in the brain hormone (10–13). The search for food and its intake in fish is girded by a series of behavioral acts matched through a supportive work between the nervous and endocrine systems (14). The control of feed ingestion behavior is a remarkable multifaceted development that comprises particularized interactions between the brain and marginal indications (15). The metabolic sensors located in the central nervous system of fishes provide room for the hypothalamic systems to receive nutritional information, allowing a qualitative control of food ingestion (16). The neural effectors of the hypothalamic origin facilitate the control of food consumed by the fish, thus, by integrating between hunger and satiety signals (17) which include apelin and neuropeptide Y for hunger hints (18, 19), and amylin and cocaine-and amphetamine-regulated transcript for satiety hints (20, 21). As important as it is, it interests more fisheries and aquaculture firms in curbing fish growth and reproduction by changing food and/or endocrine settings.

Fish feeding behavior is miscellaneous and has been broadly examined in both wild and farmed fish from their ecological perspectives (22, 23) whiles behavioral responses of fish to feeding have been associated with feeding approaches, feeding habits, feeding regularity, feed detection mechanisms and feed preferences (24). Feeding behavior and its regulation in fish comprises of external and internal environment information being analyzed by signaling molecules and receptors in the fish. Thus, the hypothalamus, assisted by other brain sections in the fish, integrates inbound indications (3, 14). As ascertained by Volkoff and colleagues, changes in dietary behavior and cravings are frequently related to changes in gene expression and/or protein content of the appetite regulators or their receptors. That is to say, changes in the mRNA/protein levels of a given hormone due to starvation or feeding have the probability of reflecting its physiological role in regulating feed ingestion. Nevertheless, it should be noted that, in the view of the multifactorial character of food regulation, there is a probability for compensative mechanisms in the manner or conduct of feeding to take place whiles fluctuations in available hormones might not essentially suggest variations in feed intake (1).

### 2.1 Dietary Selection and Preference in Fish

Fish do not consume all the food items they come across. Dietary selection has been broadly explored in mammals, which lessen their consumption of an imbalanced diet to avoid negative dietary impacts (25). Nourishment choice is based on the preface that animals, such as fish, have “quality dietary insight” and hence, select a diet that optimally restores a metabolic imbalance as a result of a nutritional challenge (26). Fishes are selective in the choice of food that contains the necessary nutrients for their survival, growth, and reproduction. This insinuates that fishes as in other animals have evolved from extraordinary diversity of means and challenges, being able to sense nutrients and the existence of precise hungriness to regulate the intake of specific nutrients (27). The source of nutrients can be recognized by gastrointestinal receptors during food digestion, as they are released interiorly in the stomach and pass into the digestive tract. Those receptors would trigger neural action and hormonal signals that would direct brain centers about the dietary properties of food and subsequently alter feeding behavior (28, 29).

**Figure 1:**
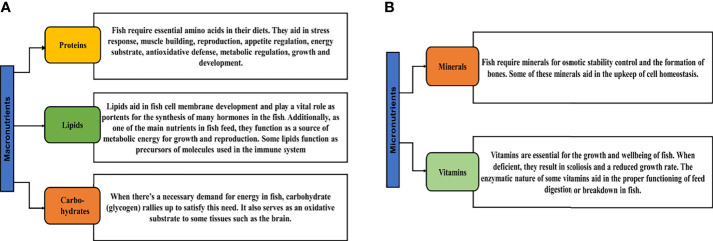
The role and importance of key macronutrients **(A)** and micronutrients **(B)** in fish food. Proteins; (9, 32, 35), lipids; (9, 36–38), carbohydrates; (8, 39, 40), minerals; (9, 41) and vitamins; (41).

Nutrition is a very significant inward factor in fish, as the foremost determination of fish feeding is to satisfy the protein and energy requirements of the fish, relating to feeding behavior and feed consumption, and have the probability to unswervingly influence or impact the appetite of the fish, reflecting in its growth (30). Nutrients are biological composites involved in biochemical reactions that produce energy and are ingredients of cellular biomass (31). These nutrients have been categorized into two groups; macronutrients and micronutrients. Macronutrients are those that are required in comparatively large quantities since they are the key source to generate the energy required by organisms to live, grow, and reproduce (32). Micronutrients on the other hand are those needed in smaller quantities, even though they have several significant roles in cellular processes (33, 34). The figure below gives examples of key macronutrients and micronutrients and their importance in feed (**Figure 1**). Detailed information on feed and nutrient requirements for fish can be found in a book from the National Research Council (NRC) (34).

The quality of feed suggests the nutritional efficacy and the objective components of a feed making it pertinent to eat and digestible for the fish (42). Fish growth, good health, and maintenance are achieved only when the precise quantity of energy and vital nutrients are available in their adequate proportions in the feed, aiding in proficient feed intake (43, 44). Response to feed intake, its tastiness, and digestibility differ as a result of the difference in fish feed components (41). Signifying that, fish farmers should access the quality of feed they provide for their fish since it plays a key role in it being accepted by the fish, how appetizing it is, and its digestibility. Similarly, the composition of a diet is an additional nutritional aspect of a feed that needs quality attention since it influences appetite-regulating hormones. While the literature available on this is insufficient as stated by Bertucci and colleagues, several research studies have it that, when macronutrients are changed in the diet composition of the fish, it has a significant impact on either the secretion and/or the expression of appetite-regulating hormones. Thus, it’s of great importance in fisheries and aquaculture since fluctuating diet and/or hormone milieu influence fish growth and reproduction (9).

Aside from the use of the ‘feed intake’ method which has extensively been used in animal nutrition, the use of ‘self-feeders’ in diet selection could be used as a great means to improve the understanding of the physiological approaches towards feeding behavior in fish. For example, studies on rainbow trout (*Oncorhynchus mykiss*) revealed that it could differentiate between diets varying in the vital amino acid content. It also showed that the fish had a precise preference for the whole diet over the balanced amino acids (45). Also, research conducted on self-selection of diets in sharp snout seabream (*Diplodus puntazzo*) and the Senegalese sole (*Solea senegalensis*) revealed that these species select macronutrients according to their dietary needs (29, 46).

According to da Silva and colleagues (27), there are numerous benefits to offering animals a free choice of nourishment, which is considered the most biological and moderate way of providing feed for fish. With time, fish can learn to select specific feeds following their nourishing requirements, as well as self-feed (46, 47). Equally, when the fish meets its goal of each specific nutrient consumption, it will provide its body with the optimal concentrations of nutrients required for proper growth and reproduction (26, 48).

### 2.2 Extrinsic Factors Influencing Feeding and Feeding Behavior in Fish

Generally, hunger stimulates the behavioral response of feeding fish. When feed is available, fish may initially feed at a faster rate and slowly decrease or stop with a gradual decline of appetite. Feeding behavior despite being influenced by intrinsic factors is extremely influenced by ecological or extrinsic factors (1, 49, 50). Below, we highlight some of these environmental factors that influence food ingestion and feeding behaviors in fish.

#### 2.2.1 Stress

Stress has been defined as the disturbance of physiological or biological mechanisms due to internal and external factors, which are generally designated as stressors (51). These provoke a cataract of consistent behavioral and biological rejoinders in which a living organism makes efforts to reestablish homeostasis, consequently incapacitating the threat. In an aquaculture firm, cultured fish are restricted, captured, crowded, sedated, held, and transported during repetitive husbandry (51). In consequence, all these taken into consideration are ordinary events in fish farming and they are possible stressors that interrupt the behavioral and biological mechanisms of the organism. Thus, causing a functional response crucial to recover the dynamic consistency (52). Reduction in feed ingestion has been described to be distinctive behavioral feedback to stress in fish (53, 54). Undeniably, stress can also disrupt several feeding conducts in fish, including the food search, finding, or capturing prey (16, 55, 56), leading to a decline in growth in several fish species (54). Fish under stressful conditions as compared to unstressed fish eat less and have slow growth. Even when food intake levels are maintained in fish, these conditions are known to persuade a decline in the conversion efficiency of feed consumed, leading to the decreased growth rate (57, 58). For example; a research by Lee and colleagues revealed that acute physical stress caused by cleaning once or thrice a week reduced the daily and cumulative feeding levels and feed conversion efficiency significantly in the sea bass (*Dicentrarchus labrax*) (59). Furthermore, these stressors have been known to adjust the control of endocrinal growth alliance in fish such as the secretion of pituitary growth hormone, among others (60–62). In the two subsections below, we discussed two key stressors that influence the well-being of fish, which needs keen attention.

##### 2.2.1.1 Temperature

**Figure 2:**
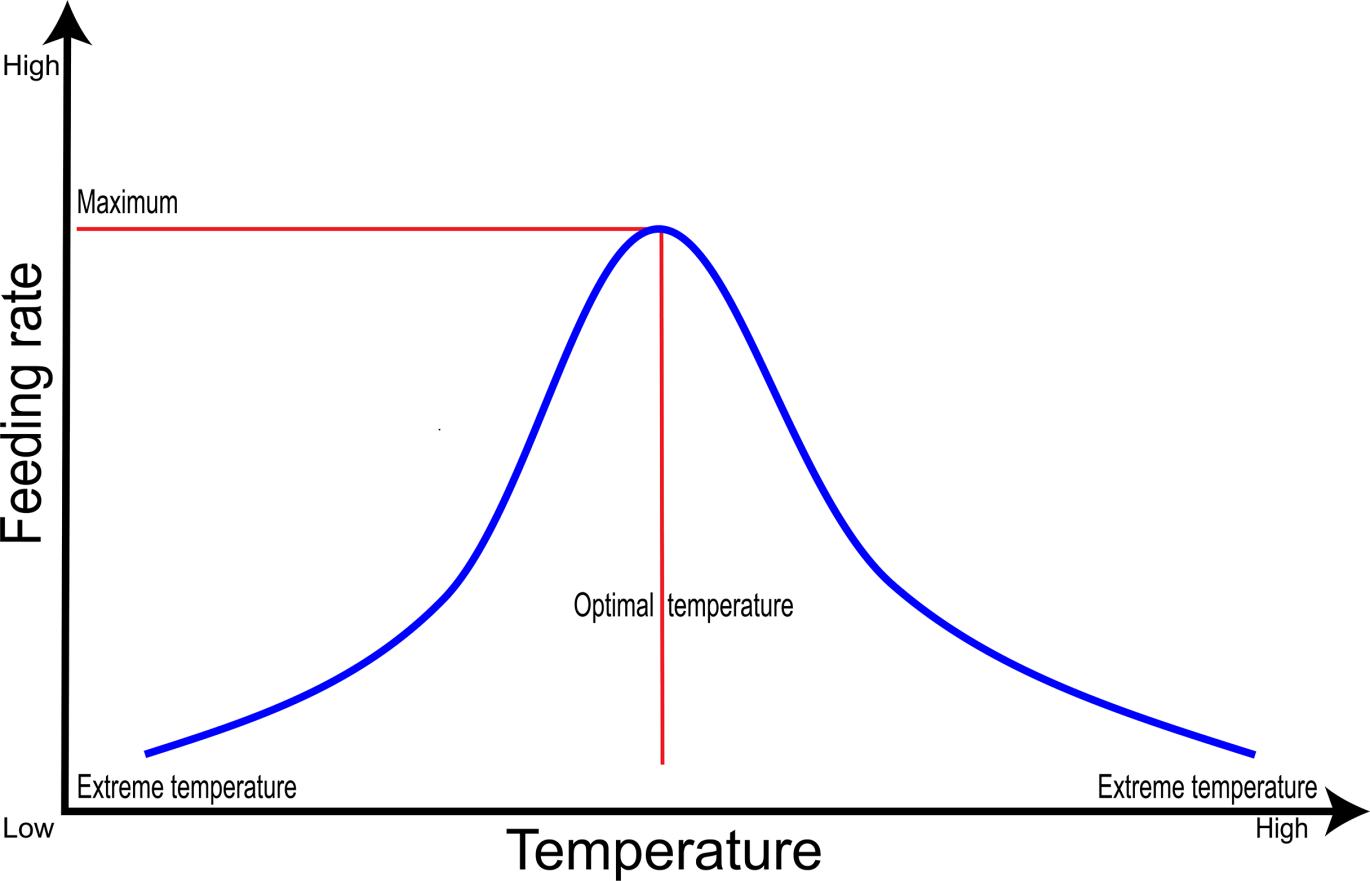
A general relative relation between feeding rate and temperature of fish species. The feeding rate decrease and subsequently stops at higher or lower temperatures (extreme temperatures).

There have been several demonstrations of the relationship that exists between temperature and feeding in several fish species. Temperature is one of the most dominant factors influencing some key biological functions in fish, including feed ingestion and feeding behavior (63, 64). Relatively, despite the complex and species-specific effects of temperature in fish, the relation between feeding/feeding behavior and temperature is like a bell-shaped structure (65); at normal temperature conditions, the voluntariness of food intake also increases (65) and/or is maintained during the acclimatization period of temperature which is specific to a particular species. On the other hand, when there’s a slight decrease in temperature, the fish adapts to the temperature and maintains its feeding rate for a short period. It has also been ascertained that before the ultimate maximal/minimal critical temperature for a species reaches, it will lose appetite, cease, and lastly stop feeding (66); see **Figure 2**. Examples given here revealed that, a research conducted on Atlantic cod (*Gadus morhua*) revealed that, when kept in a water temperature of 2°C for four weeks, there was a decrease in feed consumption compared to those kept in 11°C and 15°C water temperature (67). Also, research conducted on the red-spotted grouper (*Epinephelus akaara*) revealed that when the water temperature is around 25°C, there’s an increase in its feeding and digestion level (68). However, it should be taken into consideration that when the optimal temperature of a particular fish species reaches and/or exceeds, it results in a gradual decline in feeding behavior (69, 70).

##### 2.2.1.2 Hypoxia

Dissolved oxygen (DO) is among the most significant extrinsic factors in fish farming (71). It is known to be a key restrictive factor in aquaculture with the particular reason for the circumstance being that, fish have aerophilic absorption which requires DO at efficient levels (72). The depletion of DO concentration (hypoxia) in water bodies has been identified to be a stern extrinsic stress, which commonly occurs in high-density aquaculture (73). Reports have indicated that growth, survival, behavior as well as other physiological activities of some fish species are highly influenced by different degrees of hypoxia (72, 74) and is also known to be an endocrine disruptor (75).

Fish under severe hypoxia conditions experience reduced movement or feed intake to conserve energy (72). In research conducted on the Atlantic salmon (*Salmo salar*) with regards to the hypoxic period and its physiological activities, results revealed that there were behavioral changes associated with oxygen shortage and physiological stress in some groups. Also, the severity of hypoxia reduced the intake of feed in the fish (76). In a research study on tilapia (*Oreochromis niloticus*) it was discovered that fish kept in hypoxic conditions had significantly reduced feed intake, survival rate, and weight gain (71). Additionally, a research study on rainbow trout (*Oncorhynchus mykiss*) demonstrated that hypoxia reduces feed consumption, growth rate, oxygen consumption, energy, and lipid contents (77). On the other hand, research conducted on tilapia (*Oreochromis niloticus*) comparing three DO (normal, low and medium) levels showed that, the final fish weight of those in the normal DO levels group were significantly higher as compared to those in the low and medium DO levels groups. Additionally, fish under the low DO level group demonstrated a lower feed intake rate. The low DO level group also revealed that fish in this group had a lower growth and feed utilization rate (78). This significantly demonstrates that when fed under enough DO levels, fish show good efficiency of their feed intake (79), which will most importanlt aid in good feed conversion ratio, fish growth and reproduction in the absence of any other stress. Hypoxia has been discovered to persuade primary, secondary, and tertiary stress responses in fish (80, 81). However, most fishes can adapt to the variations in DO levels but if severe hypoxia remains, fish will sooner or later die (82).

Cultured fish always face repetitive and chronic hypoxia stress especially from overcrowding which they can barely escape due to their confined environment. Therefore, it is suggested that DO levels should be checked and highly maintained near the saturation level. In doing so, it enhances feed intake, feeding behavior, fish growth as well as improves the overall wellbeing and performance of the fish, as the result of hypoxia on the biological or metabolic actions of farmed fish would be negatively affected. A deep look into an article by Abdel-Tawwab and colleagues (72) gives more insight into the effects of hypoxia on fish growth and physiological activities.

#### 2.2.2 Photoperiod and Light Regime

Photoperiod has been known to influence and manipulate some biological functioning in fish (83). Research conducted on several fish species have revealed that photoperiod and the light regime influence their feeding activities. Photoperiod plays a significant part in the growth and survival of fish, thus influencing its feed intake and feeding behavior (84). It is known to have the ability to affect the general wellbeing and routine of fish (85).

The requirements of photoperiod and light concentration in fish are species-specific and differ for the several developing phases (84). Consequently, whiles this could be related to fish species specificity, when photoperiod is appropriately applied, it may aid in an advanced performance of the fish, thereby improving the productivity and sustainability of aquacultural practices. For example, a research study conducted on catfish (*Clarias gariepinus*) fingerlings cultured under three different photoperiod conditions; 24 hours (hrs) darkness, 24hrs light and 12/12hrs darkness and light revealed that those cultured under 24 hrs of darkness had significantly highest feed intake, best feed conversion ratio and lowest quantity of uneaten feed as compared to those cultured under 24hrs light and 12/12 hrs of darkness and light (85). Also, in a research study on the pacamã catfish (*Lophiosilurus alexandri*), it was revealed that 24hrs continuous light led to the highest feed intake (86). Going more further, in research conducted on the sharp-snout seabream (*Diplodus puntazzo*) it was concluded that although feeding behavior was strictly diurnal, 97% of feed demands were made during the light periods (87). A detailed look into how photoperiod affects fish species feed intake and feeding behavior will be of much importance.

#### 2.2.3 Circannual and Circadian Rhythms

All these external factors that impact the feeding behavior in fish have periodic or recurring styles. Thus, they affect food intake unswervingly *via* cyclical and or 24-hourly rhythms (88) or ramblingly through rhythms in endocrine systems (89, 90). All animals, even fish, showcase natural behavioral rhythms, including the two principal feed intake rhythms in fish; the daily (circadian) and seasonal (circannual) rhythms (23, 91, 92).

Several organisms including fish, exhibit annual rhythms in physiological and behavioral factors, such as feeding, reproduction, body weight, hibernation, and movement. These factors are controlled by oscillations in the secretion of hormones. The timing of these annual rhythms is delimited by changes in day length, photoperiod, or temperature, which makes available a reliable and predictive indicator of seasonal changes in environmental conditions (93). The circannual (seasonal) rhythms in vertebrates (fish) associate meticulously with ambient environmental factors, thus environmental (water) temperature and the length of the day. During the spring and summer seasons, when the days are longer and the temperature of water bodies is higher, several fish species increase their feed intake as well as their feeding behavior (23). There is limited information on how these seasonal or circannual rhythms influence feed ingestion and feeding behavior in fish, making it complex to give a straightforward conclusion about feeding activities and associated seasonal changes. As it stands now, we recommend that more research be conducted on fish feeding and feeding behavior regarding the impact of the circannual rhythms.

The circadian rhythm is a natural rhythm that is regulated by a biological daily clock that proceeds in a steady setting. This biological clock is a 24-hour cycle in the biochemical, physiological, or behavioral processes of a live organism geared for maximizing cellular activities and recognizing solar day-related environmental obstacles (94). The 24-hourly rotations of behavior and physiology (example; feeding activity) have been established in all classes of craniates, including some fish species (95, 96). Several inward or endogenic clocks prompt these circadian cycles. They consist of an independent transcriptional-translational response grummet that encompasses the recurring circadian-regulative genes expression (97) and perseveres under continuous extrinsic circumstances, such as photoperiod (98).

As in many animals, fish species consume meals at specific times during the day or night. That is to say that in fish circadian rhythms, the natural daily food ingestion times differ among species (90). Some classify specifically as daytime feeders such as Atlantic salmon, *Salmo salar* (99), redbelly tilapia (*Tilapia zillii*) (100) rohu (*Labeo rohita*), and common carp (*Cyprinus carpio*) (101) whiles others are described as night time feeders, example; European catfish (*Silurus glanis*) (102) and Zebrafish (*Danio rerio*) (103). Additionally, several fish species have showcased ideal times of eating daily (day or night). For example, research studies conducted on goldfish (*Carassius auratus*) and rainbow trout (*Oncorhynchus mykiss*) respectively revealed that the intake of food and the composition of the body is influenced by the time a single daily meal is delivered (104) whiles rainbow trout (*Oncorhynchus mykiss*) fed during their habitual or natural eating times have higher feed efficiency (105).

There are approximately a handful of known genes or hormones which regulate feed intake in fish species, including neuropeptide Y, peptide YY, ghrelin, galanin, apelin, among others. These appetite-regulating genes influence the intake of feed in two ways; feed intake inducer or inhibitor. The appetite-inducing hormones persuade or signal hunger in fish, thus causing them to search for food to eat (orexigenic factor). On the other hand, appetite-inhibiting hormones are the hormones in fish that signal their satisfaction (anorexigenic factor). Several external and internal factors affect the display of this physiological role in feed intake regulation in fish with regards to their specificity (106). As such, these factors regulate the roles of the gene either by playing opposite roles or not affect the fish at appropriate times (50). Below, we elaborate more and present summaries of the results of research findings on apelin as an appetite-regulating hormone in fish.

## 3 Apelin and Its Physiological Role in Regulating Feed Intake in Fish

### 3.1 Isolation and Characteristics of Apelin

Apelin is a 36-amino acid (AA) peptide that was initially isolated from bovine stomach extracts (107). It is a recently discovered peptide known as a ligand for the APJ receptor, a putative receptor protein related to the type-1 angiotensin receptor, and a member of the family of seven transmembrane domains G-protein-coupled receptors (GPCRs) (108, 109). From the findings of researches conducted by the research teams of Langelaan and Malyszko, it revealed in mammals that, a 77 AA precursor, prepro-apelin, gives rise to numerous forms of apelin, which can be composed of 13–36 AA residues (110, 111), thus 36, apelin-17, and apelin-13 (112).

The apelin receptor, also called APJ or angiotensin receptor-like-1 which is currently known as the common receptor for apelin was primarily cloned in 1993 due to its robust sequence homology with the angiotensin II receptor (AT1) (54% in transmembrane spheres and 31% for the complete sequence) but APJ does not bind angiotensin II (113). It is known to be an orphan G-protein-coupled receptor that was originally secluded from a human genomic collection using the polymerase chain reaction (PCR) (113). Apelin which was originally described as an endogenous ligand for APJ as stated by (107) secreted as a 77 amino acid forerunner, prepro-apelin, which is differentially processed producing numerous smaller peptide fragments, which comprises apelin-12, apelin-13, apelin-17, and apelin-36 (107, 114, 115).

### 3.2 mRNA Expression of Apelin in Fish Tissues

Several research studies have revealed the presence of the apelin gene (*apelin*) in several tissues of some fish species such as the goldfish (*Carassius auratus*) red-bellied piranha (*Pygocentrus nattereri*) and cunner (*Tautogolabrus adspersus*) these include different brain regions such as the ladder, optic tectum/thalamus, olfactory bulbs, and the hypothalamus. It can also be found in the pituitary, as well as the peripheral tissues in the fish; spleen, kidney, liver; muscle, brain, gut, gonad, gill, and heart, with seemingly higher expression levels in spleen, kidney, brain, gonad, gill, and heart (19, 116, 117). Although weakly expressed, *apelin* was also identified in the hepatopancreas, eye, intestine, and skin of the Ya-fish (*Schizothorax prenanti*) (118). Also, in *Schizothorax davidi*, apelin mRNA was expressed in the spleen and heart, considerable levels in the brain (myelencephalon and telencephalon), liver, and trunk kidney (119), and pirapitinga, *Piaractus brachypomus*, *apelin* mRNA expression was revealed in the liver, stomach, pyloric caeca, foregut, hindgut, kidney, gill, skin, and muscle as well as in the brain and pituitary (117).

### 3.3 Non-Appetite Regulatory Role of Apelin

Apelin is known to control cardiovascular functions in mammals, including blood pressure and blood flow (109). The apelin/apj system plays important and several roles in the physiology and pathophysiology of many organs, including the regulation of blood pressure (120), cardiac contractility (121, 122), among others. It is known to be one of the most effective stimulators of cardiac contractility yet discovered and plays a role in cardiac tissue renovation in vertebrates (123–125).

### 3.4 Apelin as an Appetite-Regulating Hormone in Fish

Apelin, which has an uncertain role in the regulation of feeding in mammals is known to act as an orexigenic factor and might have several biological regulating roles in fish (19, 119, 126). Either its peripheral and/or central injections increased food intake in fish species that have been studied. For example; research conducted on the blind cavefish, *Astyanax fasciatus mexicanus* revealed that peripheral injection of *apelin* significantly increased food intake of the fish as compared to saline injections (127). In goldfish (*Carassius auratus*) both intraperitoneal (i.p.) and intracerebroventricular (i.c.v.) injection of *apelin-13* revealed an augmentation of its food intake (19). Also, in the Siberian sturgeon (*Acipenser baerii*) continuous i.p. injection of *apelin* demonstrated an increase in feed ingestion (126). Additionally, *apelin* i.p. injection in Ya-fish (*Schizothorax prenanti*) also stimulated the intake of feed (118).

### 3.5 The Response of Apelin to Fasting and Refeeding in Fish

Several hormones in fish as in other vertebrates control the intake of food. These hormones, known as appetite-regulating hormones are produced from the brain and or other marginal tissues in the body of the fish (50). These appetite-regulating hormones play roles either as a food inducer or inhibitor. Apelin’s role on food intake in vertebrates like teleost is poorly understood (118). Nonetheless, researches have been conducted to find out the role that apelin plays in some fish species’ food intake, either as an orexigenic or an anorexigenic indicator. Here, we elaborate more on its response to feeding and fasting, indicating which role it fits well into.

Research conducted on common carp (*Cyprinus carpio*) discovered that starvation resulted in a significant upsurge in hypothalamus *apelin* and expression of *APJa* mRNA. It then returned to normal levels after the fish were refed. Also, the expression of *APJb* mRNA augmented after temporary starvation, thus within 2 and 4 days; nevertheless, there was no significant difference between fed fish and refed fish even after the starvation was prolonged. From this same research, there was a significant increase in *apelin*, *APJa*, and *APJb* mRNA expression levels in the foregut of the common carp, which then returned to normal levels after refeeding, either after a short-term or long-term fast (128). It was discovered from a research study that the abundance levels of *apelin* mRNA were greater in starved goldfish than in fed goldfish (*Carassius auratus*) in both hypothalamus and telencephalon (19). Another research study also revealed that *apelin* mRNA levels in the whole brain were higher at 1 hr after feeding than that of unfed Siberian sturgeon (*Acipenser baerii*). However, its expression returned to normal levels at 3 hrs after feeding (126). In this same research study, it was revealed that *apelin* has bidirectional effects on feeding regulation in the Siberian sturgeon (*Acipenser baerii*) thus, *apelin* acts as a satiety factor in the short-term feeding regulation and a hunger factor in long-term feeding regulation (126). Moreover, in research conducted on *Schizothorax davidi*, it was concluded that *apelin* expression of fed fish at + 1 hr and + 3 hrs after feeding was lower than that of unfed fish, and *apelin* expression in the hypothalamus of unfed fish augmented on the 5th and 7th days and when fasting fish were re-fed, *apelin* mRNA expressions disclosed a notable decrease from the 9th to the 14th day concerning the fed group (119). Furthermore, in the red-bellied piranha (*Pygocentrus nattereri*) fasting induced a significant increase in the mRNA expression of *apelin* in the brain (129).

Research conducted on Ya-fish (*Schizothorax prenanti*) revealed that there was about 2.5 and a 2-fold decrease in *apelin* mRNA expression in the hypothalamus of fed fish at 1 hr and 3 hrs post-feeding compared to unfed controls, respectively (118). Both the levels of *apelin* and *APJ* mRNA expressions had a decreasing trend hours before feeding. In this same research, concerning food deprivation, food-destitute Ya-fish (*Schizothorax prenanti*) had a noteworthy change of about 1.8-fold higher mRNA expression levels of *apelin* than 3, 5 and 7 days habitually fed controls. The mRNA expression of *apelin* was significantly decreased when 7-day fasted Ya-fish (*Schizothorax prenanti*) were re-fed, and the levels of the 7-day fed control group and fasted group of Ya-fish (*Schizothorax prenanti*) had an about 1.5- and 2.5-fold higher than the 7-day refed fish, respectively.

To sum up, the role of apelin is highly attributable to appetite regulation in fish. That is to say, the apelin hormone in starved or unfed fish induces hunger (up-regulated) and thus, persuades the fish to go after or search for its meal and there’s a gradual or complete decrease in the expression of *apelin* as the fish eats or post-eating. A referral to **Figure 1** in an article publish by Assan and colleagues (50) gives a clear clue on the existing relationship between appetite-inducing genes and appetite-inhibiting genes.

**Table 1** summarizes fish that have been used as models for *apelin* research studies as appetite-regulating factors.

**Table 1:** Summary of fish used as models to identify apelin as an appetite-regulating hormone.

Fish models	Treatment	Duration of treatment	Gene regulation	Tissues with the highest mRNA expression	Reference
Ya-fish (*Schizothorax prenanti*)	Starvation	7 days	Up-regulation	Heart, spleen, hypothalamus and kidney	(118)
Common carp (*Cyprinus carpio*)		8 days		Brain, pituitary gland, spleen, and kidney	(128)
Goldfish (*Carassius auratus*)		7 days		Spleen, kidney, brain, gonad, gill, and heart	(19)
Siberian sturgeon (*Acipenser baerii*)		15 days		Brain, spleen, stomach, and kidney	(126)
Red-bellied piranha (*Pygocentrus nattereri*)		7 days		Spleen, kidney, heart, and brain	(129)
*Schizothorax davidi*		14 days		Brain, heart, spleen, liver, and trunk kidney	(119)

### 3.6 The Interactions of Apelin With Other Appetite-Inducing Hormones in Fish

Recent researches have demonstrated that individual orexigenic molecules or hormones interact with each other. For example, in cavefish, *Astyanax fasciatus mexicanus*, *apelin* i.p. injections increased *orexin* brain expression but did not affect either *cholecystokinin* or *cocaine- and amphetamine-regulated transcript* expression, suggesting that *apelin* might increase food intake through the stimulation of the orexin system in cavefish (127). Additionally, it was demonstrated by *in vitro* and *in vivo* experiments that *apelin* could persuade important mRNA expression levels of appetite-related and growth-related genes, including *neuropeptide Y*, *agouti-related peptide*, and *orexin*. This suggests that *apelin* has the potential to control the food intake and development of common carp by regulating the expression of these vital genes (130).

### 3.7 Characteristic Similarities Within Appetite-Regulating Hormones

Besides the fish-species-specificity and other intrinsic and extrinsic factors antagonizing appetite-regulating hormones from exerting their full function on regulating feeding in teleost and other fish species, other genes play the same specific role in feed regulation in fish as apelin does. See **Table 2** for a list of other appetite-inducing hormones in fish.

**Table 2:** List of other known appetite-inducing hormones in fish.

Other appetite-inducing hormones	Key expressed tissue	Reference
Ghrelin	Gastrointestinal tract	(131, 132)
Neuropeptide Y	Brain/Gastrointestinal tract	(12, 18)
Agouti Related Protein	Brain	(12, 133)
Orexins	Brain	(12, 134)
Galanin	Brain	(135, 136)
Growth hormone	Pituitary	(137)

Generally, appetite-inducing hormones (hunger or orexigenic hormones) serve as hunger signals, causing an increase in feed ingestion. That is to say, fasting or starvation causes an up-regulation of these appetite-inducing hormones in fish, for example; *ghrelin* (131, 132), *neuropeptide Y* and *orexin* (12, 18). On the other hand, appetite-inhibiting hormones (satiety or anorexigenic hormones) cause a reduction in food intake, thus fasting or starvation does not affect their expression but rather feed intake causes an up-regulation of these appetite-inducing hormones. Thus, appetite-inhibiting hormones in fish demonstrate pre-prandial decreases and postprandial increases in their concentrations. Example; *peptide YY* (50), *cholecystokinin* (138), and *cocaine- and amphetamine-regulated transcript* (20).

It has been demonstrated that the peripheral or central orexigenic hormone injections in fish persuade a significantly increase in food consumption rate as indicated in some research studies (15, 118, 126, 127, 139). Also, experiments demonstrating the acute and or chronic effect of anorexigenic hormone injections on either peripheral tissues or the brain of some fish species revealed that there were significant reductions in food ingestion for a short period in the acute and a long period all through the whole experiment for the chronic injection. Examples of such experiments include those conducted by (127, 140).

## 4 Conclusion

Data available on fish feeding regulations indicate that the fundamental mechanisms in regulating feeding behavior are conserved. Our knowledge about how extrinsic factors influence feed ingestion and feeding behavior has been simplified. However, it appears that the general scheme of feeding regulation in fishes is similar to that of other vertebrates in the sense that hunger and feeding are controlled by central feeding centers that are influenced by endocrine factors rising from both the brain or from marginal tissues. As a whole, we believe there is still limited information available in fish compared with other organisms regarding how these extrinsic factors influence fish feeding and feeding response.

The role of *apelin* is highly attributable to appetite regulation in fish species that have been studied. To date, most researches conducted on appetite‐regulating hormones in fish species have been relatively short‐term studies, thus, making it difficult to establish a relation between short‐term and long‐term appetite‐related factors. Fish have been known to exhibit a wider range of feeding behaviors, feeding habits, and feeding adaptations, including fasting or starvation periods. Here we suggest research to be advanced on the mechanisms regulating feeding and appetite-regulating hormones and genes in fish. Also, research on the response of *apelin* to feeding, fasting, and re-feeding should be conducted based on the influence of these extrinsic factors, adding up to the existing studies.

## Author Contributions

DA designed the content and structure of the whole paper. UFM designed the figures. DA, YH, UFM, and MA wrote the review. GL and HC proofread and revised the paper. All authors contributed to the article and approved the submitted version.

## Funding

This article was supported by grants from the Key Research and Development Program of Guangdong (2021B202020002), the Guangdong Basic and Applied Basic Research Foundation (2019A1515010958 and 2019A1515012042), the Southern Marine Science and Engineering Guangdong Laboratory (Zhanjiang) (ZJW-2019-06), The Open Project of the Key Laboratory of Utilization and Protection of Tropical Marine Biological Resources (Hainan Tropical Ocean University), the Ministry of Education (grant number UCTMB20201), and Guangdong South China Sea Key Laboratory of Aquaculture for Aquatic Economic Animals, Guangdong Ocean University (No. KFKT2019ZD07).

## Conflict of Interest

The authors declare that the research was conducted in the absence of any commercial or financial relationships that could be construed as a potential conflict of interest.

## Publisher’s Note

All claims expressed in this article are solely those of the authors and do not necessarily represent those of their affiliated organizations, or those of the publisher, the editors and the reviewers. Any product that may be evaluated in this article, or claim that may be made by its manufacturer, is not guaranteed or endorsed by the publisher.
